# Discovering dignity through experience: How nursing students discover the expression of dignity

**DOI:** 10.1177/09697330211012042

**Published:** 2021-09-15

**Authors:** Tone Stikholmen, Dagfinn Nåden, Herdis Alvsvåg

**Affiliations:** VID Specialized University, Norway; Oslo Metropolitan University, Norway; VID Specialized University, Norway

**Keywords:** Dignity, hermeneutics, nursing education, nursing students, nursing students’ experiences

## Abstract

**Introduction:**

Dignity is a core value in nursing. Nursing education shall prepare students for ethical professional practice and facilitate insight into the phenomenon of dignity and its significance. There is limited knowledge about how nursing students discover dignity in their education.

**Research aim:**

The aim of the study is to develop an understanding of how nursing students discover and acquire dignity.

**Research design:**

The study has a hermeneutic approach where qualitative interviews of nursing students were employed. The process of interpretation was inspired by text of Fleming, Gaidys and Robbs.

**Participants and research context:**

Nineteen nursing students agreed to be included in the study, representing six different campuses at three different educational institutions. All were in the final year of their study. The interviews took place at the educational institutions.

**Ethical considerations:**

The educational institutions facilitated recruitment of the students who signed voluntarily for participation and continuous informed consent. The study was approved by The Norwegian Center of Reporting Data (NSD). The research recommendations of the Declaration of Helsinki were followed.

**Findings:**

The nursing students discovered the expression and significance of dignity through experiences, gained through introspection and in interaction with others during the education.

**Discussion:**

The findings are discussed using Gadamer’s concept of experience and how experiences can create new insight. In particular, the students’ experiences with the inner ethical and external aesthetic dimension of dignity are discussed.

**Conclusion:**

The study shows that students discovered the inner ethical dignity through experiencing vulnerability, pride and shame. They discovered the external aesthetic dignity through incidents, where they experienced both to be confirmed and not to be confirmed, and through observation of good or bad role models. Crucial negative and positive experiences are important for discovering the expression and significance of dignity.

## Introduction

Dignity is a core value in nursing.^1^ Both nurses^2^ and nursing students^3^ rate dignity as the most essential value in nursing. The importance of dignity is emphasized in the Code of Ethics of Nursing^4^ and is thus incorporated in the nursing education that prepares the students for ethical professional practice.^5^ The theme of this article is how nursing students experience and discover the significance of dignity.

A general belief is that dignity means having an inhabiting value.^1^ Dignity is described as a human need, a value, a collection of values, a competence or a skill. Humans can both experience and convey dignity.^6^ This shows that dignity is complex and diverse.^7,8^ However, dignity often appears as something vague and abstract, can often be taken for granted, treated superficially and be devalued.^8,9^ Due to the complexity of dignity, it is difficult to know how dignity can be expressed and how students gain insight into the phenomenon.^1,10^

Nursing students need insight into dignity.^11–13^ Research shows that if the values of professional nursing are well integrated in nursing students, this will help them develop an identity as nurses, make sound ethical decisions, alleviate moral stress and increase the quality of nursing.^14–16^ Some authors also claim that deeper insight into dignity in particular, will help students maintain their own sense of dignity and strengthen the ability to promote this value in interactions with patients.^7,11,12^

Research has been carried out on how nursing students acquire values in general and dignity in particular, but several of these studies are intended to operationalize how dignity can be learned through different techniques and learning methods.^10,17,18^ Other research indicates that dignity is an abstract inherent phenomenon in humans and something that is integrated through processes of socialization.^8^ However, others argue that the education must facilitate it so that dignity can be discovered through experiences. These experiences may then activate intellect and emotions, and students may become aware of their values and how these affect them and their nursing practice.^9,19–21^ Furthermore, research refers to the importance of lived life experience, learning culture and role models.^17,18,20,22–24^

There is a need for research that sheds light on how ethical values, such as dignity, are promoted in students.^20,25,26^ Nursing students are underrepresented in research in this field.^7,8,21^ Because research is scarce, we found it important and necessary to carry out this study.

The purpose of this study is to develop an understanding of how nursing students discover and acquire dignity. The research question is: How do nursing students discover the expression and significance of dignity during their education?

## Theoretical framework

In this article, dignity will be understood according to the description of this concept by Eriksson,^27,28^ Edlund^6^ and Edlund et al.^29^ They describe dignity consisting of an absolute and a relative dimension. The absolute dignity is constant, inviolable and non-measurable. It is about the holiness of humans, the absolute human worth. Through creation, humans are given a unique position among created beings. It involves freedom, responsibility and duty to serve one’s fellow men.^6,27–29^ The absolute dignity is expressed and confirmed through the relative dignity. Relative dignity is changeable, it can be violated and rebuilt. It is socially rooted, created and shaped by culture and society, and people experience it in relationships. It is experienced when humans experience harmony between their own abilities, knowledge and the demands they have towards themselves or others have towards them. Dignity becomes perceivable when one meets people who convey dignity.^6,29^ The relative dignity is divided into an inner ethical dignity and an external aesthetic dignity, both of which are symbols or expressions of absolute dignity. The inner ethical dignity is a psychic dimension for the experience of dignity. It can be expressed as pride, nobility, position, rank and independence. The inner ethical dignity draws attention to one’s own and others’ dignity and contains values such as morality, ethical attitude, principles and ideals. The external aesthetic dignity is a bodily dimension that is expressed in concrete actions, attributes, products and symbols. It contains values such as respect, grandiosity, properness, suitability, restraint and orderliness, and focuses on how dignity is expressed in action. When a value in the inner or external dignity is no longer attainable and the dignity is threatened, the value will be replaced by another value that can symbolize human dignity, so dignity is restored.^6,29^

## Methodology

### Design

The study has a hermeneutic approach based on Gadamer’s philosophical hermeneutics. According to Gadamer,^30^ preunderstanding is a condition for understanding. Preunderstanding is an already interpreted and organized understanding of the world, which forms the human horizon of understanding in relation to the world and the phenomenon being explored.^31^ According to Gadamer, all understanding contains an interpretation, and all interpretation is verbal. Understanding is achieved through a dialectical process. The interpretive process that provides understanding is called the hermeneutic circle. There are constant movements between the whole and the individual parts, between the phenomenon and the context in which the interpretation takes place, and between the human preunderstanding and what is interpreted. Meaning emerges after constant corrections. It is understood in the light of the context from which it springs, and the parts are understood in the light of the whole.^30^

### Participants and research context

The participants were recruited from students in their final year of nursing education, from six different campuses at three different universities or university colleges in Norway. All campuses had different sizes and different curricula. The nursing education the students underwent consists of about 50% theory studies and 50% practice studies. Through theory and practice, students achieve various learning outcomes, for example, learning outcomes that ensure knowledge of key values and concepts in nursing, such as dignity. The practice studies aim at giving the students experience with their future professional roles through supervision, follow-up and assessment. The assessment focuses on the achievement of the various learning outcomes of each practice study.^5^

All the interested and qualified nursing students received written and oral information. The study had a strategic accessibility sample, and the recruitment led to a sample of 19 nursing students, 15 women and 4 men, aged between 21 and 37 years. The students signed a consent form. Everyone who satisfied the inclusion criteria and wanted to join the study was included.

### Data collection

Interviews were conducted with nursing students, where the goal was to obtain descriptions of their experiences with dignity within the nursing education. We used a semi-structured interview guide with flexibility of discussion themes regarding the order of topics. Examples of questions are the following: What do you understand by dignity? How do you learn dignity? Efforts were made to create a safe and generous atmosphere, where the researcher was listening, encouraging and affirming. The conversations flowed easily, and the atmosphere was good. Nonverbal observations were noted. On average, the interviews lasted 76 min.

### Data analysis and interpretation

The interviews were recorded and transcribed verbatim. Both the situations and the contexts in which the interviews took place, as well as the transcribed text, were interpreted. Gadamer does not present a specific method of analysis, but his ideas on how understanding is achieved have been good tools in the interpretation process. In addition to Gadamer’s^30^ thinking, the process of interpretation has been inspired by Fleming et al.^32^

Efforts were made to raise awareness of the hermeneutic situation throughout the research process. Researcher’s preunderstanding was recorded and made available for reflection.

The audio files were listened to several times, and the transcribed text was read repeatedly to create an overall impression of the text. The overall impression was summarized in both figurative and textual form and was the basis for the interpretation process. The whole affected how the various parts of the text were understood.

In the interpretation process, questions were asked of the text, such as what is the expression of dignity here and how do students discover dignity? Meaning units were identified and themed. They were further put together with related themes from all the interviews and read collectively to look for different nuances within each theme. Shades of themes were divided into different sub-themes. The research team discussed possible themes and sub-themes, and how they were connected. Throughout the process, the various parts (theme and sub-theme) were mirrored against our own preunderstandings and the overall impression. The understanding of the whole was expanded, which in turn gave a new understanding of the various parts. The process of interpretation led to two main themes about how students discover the expression and significance of dignity (Table 1).

**Table 1.** Example of the interpretation process.

Overall impression	Meaning units	Sub-themes	Main theme
Personal experiences	‘I think of dignity as something very personal. It relates to the person and human in itself and on the respect you get. That definition makes me very vulnerable. Especially considering that we use ourselves in practice and get feedback on how we are, it makes me very vulnerable’. (9)	Vulnerability	Dignity – through introspection
	‘You care about yourself and your self-appearance. I believe this relates to dignity. […] For me, it’s about taking pride in the work I do, the person that I am and to what extent I can manage on my own and be independent’. (10)	Pride	
Experiences in relation to others	‘I have gained a different awareness about dignity. […] Through new experiences. For example, patient situations that have made me think’. (5)	Patient meetings	Dignity – through interaction
	‘The clinical supervisor has been a very good role model and have shown me how to promote patients’ dignity’. (3)	Different role models	

### Ethical considerations

The Declaration of Helsinki^33^ on voluntary participation and continuous informed consent was followed. The nursing students were informed that participation in the study was voluntary and that if they withdrew from the study it would not have any consequences for their further studies. It was also informed that the researcher was not affiliated with any of the educational institutions at which the students studied. Throughout the research process, it was reflected that the conversations were affected by an asymmetric power relationship. Some students found it challenging to talk about their own vulnerability. Therefore, in some cases, the students were contacted afterwards to make sure they were doing good. Possible strains for the students were assessed against what benefit the study could provide and what new insights and experiences the students were left with after contributing. The requirement of confidentiality was met by deidentifying the audio tapes during transcription. The students’ names were numbered from 1 to 19. Audio files, consent forms and wiring keys were properly stored in a lockable cabinet. The study was approved by The Norwegian Center of Reporting Data (NSD), and current guidelines were followed.

## Findings

The nursing students discovered the expression and significance of dignity through experiences during their education. The experiences are interpreted in two main findings: (1) dignity – through introspection and (2) dignity – through interactions.

### Dignity – through introspection

The students made many discoveries when they entered into the student role. First, the students got to know themselves in a new way. Through their new experiences in the education, they discovered their own vulnerability and how this vulnerability made them aware of dignity, the importance of dignity and the need to come up with a response to maintain their own dignity. Experiences with their own achievements gave the students a sense of pride or shame. In the space between pride and shame, the vulnerability of dignity was discovered.

#### Vulnerability and its response

During their education, students gained experiences with their vulnerability. Several were in the early stages of adulthood, lived alone for the first time, uprooted from familiar surroundings and close relationships, and felt lonely and fear of not making friends. Some had to deal with their own illness, as well as illness and death in close family. Some were dealing with relationship break-ups and were single parents, while others had immigrated to Norway and struggled with both language and cultural barriers. Several described life as vulnerable and demanding:

You are kind of vulnerable all the time. (16)

The students found the education to be demanding, and when facing the requirements and expectations they felt vulnerable. Previous learning strategies were no longer useful, and the transition from high school was considerable. They had painfully experienced the complexity and breadth of the nursing profession. Students who had done well in high school had for the first time problems with ‘keeping their heads above water’, and several had struggled with the feeling of inadequacy during their studies. They described becoming a nurse as getting through ‘an eye of a needle’, where there was no room for mistakes:

There is never room for having a bad day. It is not permitted to not be switched on. (10)

During the clinical studies, students experienced themselves at their most vulnerable. The clinical studies were perceived as busy and tiring. For many, it was the first encounter with patient’s illness and suffering. The students saw themselves as visiting and not as part of the community. It was difficult for them to be constantly new and inexperienced and show their vulnerability:

You are exposed as completely ignorant. And then you come to a specialized ward where they are so competent, and you struggle to locate where the organs are. (2)

Increasing awareness of their own vulnerability made students aware that their dignity was at stake. The students needed to express and protect their own dignity when faced with their vulnerability. The students needed to find a ‘token’ for themselves that could represent their dignity and thus remedy the vulnerability they felt. They found different ‘tokens’, but the common denominator was something positive that could represent their own dignity:

I have always received good grades, great point average in middle school, good grades in high school. If I came home with a top grade and told the others, it was like; we knew it! (5)

The students highlighted situations where they had shown great capacity, performed well under pressure, mastered and shown different forms of competence. They were in pursuit of excellence and longed to show their infallibility and perfectionism. They wanted others to see and understand them as the people they wanted to be, namely, good people with good values, attitudes and competence. This became especially important in encounters with patients:

I think dignity is related to caring about your appearance. Taking pride in the work I do, and in the person, I am, and to what extent I am independent. (10)

#### Pride and shame and the space between

The students described dignity as something personal. This made them vulnerable. They believed that becoming a nurse required character and special personal qualities. Therefore, they had great concern about underperforming, and by not mastering, it was not only their abilities that failed, but they failed as human beings. The defeat was personal, and the experience of dignity was absent:

It’s so personal, in a way. You use yourself. And when you fail, it is you as a human being that fails. […] So, when you use yourself, you become vulnerable to criticism. Because it’s you who’s not good enough. (9)

Whether the students assessed themselves as good enough was important for their confidence. In many ways, this was the ultimate test for the students, and it left them with an experience of either pride or shame. It was all depending on how they had lived up to their own or others’ expectations. Living in the space between feeling shame or experiencing pride made the vulnerability of dignity clear. It made them more aware of their own sense of dignity and thus also the importance of it.

The students became acquainted with dignity in relation to pride. Pride was built up through experiencing mastery in situations:

Every time I complete a clinical study, I am proud and get a feeling of achievement, no one can take that from me. […] So when I master something new, it kind of becomes part of my dignity. (10)

The experience of pride was described as a feeling of victory and to suffice, as a boost of self-confidence. Lack of pride may lead to shame due to inadequacy. They were ashamed of not being able to live up to the standard they had set for themselves or the standards others had set for them:

I have a sense of guilt; I should have known better. Because I made some completely stupid mistakes. I overlooked something obvious. And then I feel stupid. This should not have happened. (5)

Shame was expressed as a feeling of being unsuccessful, useless and worthless. They described themselves as lagging behind, being below average and not good enough. They lost confidence in themselves and also believed that others looked down on them. This made them aware of dignity and the need to experience dignity in relation to others.

### Dignity – through interaction

In addition to discovering dignity through introspection, students discovered dignity in interactions with others. Dignity was experienced personally and situationally through interactions at the educational institution, in classes and supervision, and during the clinical studies. The education consisted of interactions where dignity was both conveyed and confirmed.

#### Confirmation or lack of confirmation

The students observed that they primarily gained knowledge of dignity through how their own dignity was promoted or violated during their education. What helped to determine how they experienced this was the degree of confirmation they received through interactions:

I think you discover dignity through both experiencing dignity and not experiencing dignity. (14)

Although the students found that dignity was difficult to define, they clearly recognized it as a feeling or a mood:

It’s so difficult to define dignity, but I know when something is dignified. I also recognize when dignity is lacking. I can give many examples of when I feel dignified and when I do not feel dignified. (3)

Experiences, positive or negative, were experienced bodily. It could be emotions such as melancholia or joy, uselessness or self-satisfaction. The confirmation they received through their interactions encouraged the students’ belief in their own dignity. It gave them hope and helped them to perform better. But it also left them at the mercy of the people they met. The encounters could make all the difference in how they experienced themselves and their education. Examples of confirmations that in a special way were important for the students’ sense of dignity and discovery of dignity were encounters with fellow students, patients, clinical supervisors and nursing teachers. When interacting with fellow students, they experienced a lot of confirmation of their own dignity. They discovered dignity through well-being, good relationships and a good learning environment. In encounters with patients, dignity was recognized through the experience of being important to others. Regarding clinical supervisors and nursing teachers, the students experienced that their dignity was at stake. The experience could vary from fantastic to completely destructive, depending on how they perceived the relationship and its content. For example, students experienced dignity when they were cared for, valued and respected in the relationship. Relationships where students experienced being overlooked, ignored and degraded hampered the sense of dignity. The students wanted to tell positive stories of dignity but stated that it was easier to identify dignity when it was violated:

If you have first experienced that feeling of having no dignity, you become aware of it. I am very aware of it now. (4)

#### Good and bad role models

The students were aware that it is the nurses’ duty to promote patients’ dignity according to laws and guidelines, but they called for help to understand how this should be done in practice. Therefore, it became important for the students to observe how others managed dignity in practice. In particular, the students observed how nursing teachers and clinical supervisors either promoted or violated their or the patients’ dignity:

I have learned a lot from seeing in practice how the nurses interact with patients, how they behave around them and talk to them. I think they are good role models. I have them in mind when I think about how I want to be as a nurse. (7)

Based on what they observed, the students ranked nursing teachers and clinical supervisors as either good or bad role models. The students believed that dignity had to be evident in order to be valid. It became real and tangible only in a specific situation. How the persons managed to realize dignity determined how the students rated them as role models. The students used themselves or their loved ones as a benchmark. When being with patients, they assessed the situation and asked themselves how they or their loved ones would experience the situation.

The nursing teachers and clinical supervisors that the students believed promoted dignity through interactions became good role models who helped to shape them and how they themselves wanted to become as nurses. The students believed that good role models were about the individual nursing teachers’ and clinical supervisors’ personal characteristics, attitudes and behaviour. They were described as available, attentive, confident and proud nurses:

There is a teacher here that we are talking about. She is a proud nurse. […] There’s just something about how she talks about her occupation. I remember one time when she was lecturing, she started crying because she told a story that touched her. (15)

The students had also encountered what they called bad role models. These were difficult encounters where they reacted on behalf of the patients whom they felt did not receive dignified treatment. These people were described as negative, degrading and bullying in their interactions. The students first and foremost became aware of how dignity could be promoted by observing good role models. But they also pointed out that they discovered the expression of dignity through experiences with what they called ‘examples of horror’:

I am motivated to be more like the nurses whom I think are good. The ones I look up to and think ok; that’s how I want to be. But you are motivated by the bad guys as well. For you think; ok, at least I’m not going to be like this. It also gives motivation to do a better job. (17)

Situations where students felt that their or patients’ dignity was at stake made them stop and think again. It was contrary to their expectations. The mere experience affected them. They felt undignified having to witness this.

Through these experiences, the students recognized dignity. They highlighted that their awareness of dignity had evolved during their education. They felt better equipped to maintain both their own and others’ dignity, and the relevance of dignity increased. At the beginning of their education, the students took dignity for granted, but as they finished their education the students were more aware of the concept of dignity and had experienced its fundamental significance:

It’s a bit awkward to say, but I think differently now. I do not know if it is because I am older now, or if it is because of my nursing studies, but I think it is related to my studies here.

## Discussion

The main finding of the study is that dignity is discovered through experiences with introspection and with interactions. This will be discussed in the following using Gadamer’s^30^ philosophical hermeneutics, Edlund et al.’s^6,29^ and Eriksson’s^27,28^ understanding of dignity, as well as other research.

### Discovering dignity through experience

Our study shows that it was the students’ personal experiences with dignity through education that had given them most insight about the expression and significance of dignity – something that had become visible, touched them, made them react and reflect, and make new discoveries. According to Gadamer,^30^ understanding begins when something is discovered because the discovery is connected to our values, our experience and history. Understanding is more associated with experience rather than specific and verifiable knowledge. Something touches us because it appeals to us.^34^ The findings show that dignity was difficult to explain but was experienced when the phenomenon was present or absent. The experiences were bodily, like emotions, and they provided insight. Gadamer^30^ writes that understanding comes through experience. They are incorporated into our preunderstanding and are crucial for new understanding. For Gadamer, experience is an ‘event’, a phenomenon we encounter. We must first be gripped by a phenomenon and experience it before we understand it. The students’ experiences of dignity affected them, and they gained new understanding. New experiences lead a man into a dialogue with his own tradition, understood as his own preunderstanding or in a dialogue with the phenomenon itself. Through dialogue, one’s own preunderstanding is challenged; it can break with what one has previously understood, something Gadamer calls a negative experience. Negative experiences are real experiences because they form new insights. Something new is being revealed. Something that used to be unfamiliar becomes visible. The horizon of understanding is changing.^30^ The findings show that education is experienced differently than expected. The students had not expected their own dignity to be challenged like it did. Nor did they expect that nursing teachers and clinical supervisors did not always promote their or the patients’ dignity. The students gained a new understanding of dignity through breaches of expectations and preunderstanding.

#### Experiences within the inner ethical dimension of dignity

The absolute dignity is constant and inviolable. The relative, on the contrary, is impressionable and can be both violated and rebuilt. It is influenced by culture, society and relationships.^29^ The findings show that the students’ relative dignity was significant in the discovery of dignity. Their experiences led to introspection, which led them to be acquainted with the inner ethical dignity. As students, they had entered a new situation and been given a new role. The student role involved insecurity, estrangement, the feeling of being undressed and lowest in the hierarchy. The nursing education was experienced as demanding, and several felt that it was difficult to meet their own and others’ expectations. The inner ethical dignity serves as a symbol of absolute dignity, in the form of pride, nobility, position, rank and independence.^6^ These were symbols that the students rarely recognized. They felt vulnerable and their dignity was at stake. They no longer had anything that symbolized their dignity.

When the students perceived their dignity as threatened, it became important for them to ‘adorn’ themselves with something. They wanted to appear as infallible, proper people, with good attitudes, and as future nurses who knew their profession and who acted morally right. In this way, their dignity could be rebuilt. Edlund et al.^29^ believe that human dignity is shaped by values that symbolize human dignity. When some values no longer are achievable, these values are replaced with other values that can represent dignity. Values that replace the lost values are closer to the absolute dignity.^6,29^ Values that symbolize ‘being’ more than ‘doing’ becomes more important. The question of who they were and what they stood for became crucial for the students, rather than their abilities. In this way, they discovered the absolute dignity and came closer to the core of the phenomenon.

The inner ethical dignity relates to the psychic dimension and expresses the experience of dignity. In relation to this dimension, one becomes more self-aware and more aware of one’s own sense of dignity.^6,29^ It is important to have a personal experience of one’s own dignity, while culture and context influence the experience.^35^ The findings show that the students became increasingly aware of how they felt. They were amazed at how vulnerable they were. They no longer experienced harmony between their own abilities, knowledge and the demands they or others had on them, which is essential to the sense of dignity.^29^ The culture and the context in which the students lived required something new. Although the students tried to adapt to the new requirements, there were several who experienced inadequacy.

According to Eriksson^27^ and Edlund et al.,^6,29^ man has been given a unique position among created beings. It involves freedom, responsibility and duty to serve one’s fellow man. Being able to meet the demands and expectations that responsibility and duty entail contributes to the sense of dignity. The findings show that the students recognized that dignity is an essential value in the nursing profession, that in their future professional role they should safeguard the dignity of patients and therefore promote this value. Experience of success in education, therefore, contributed to pride in achieving the goal. Conversely, the feeling of inadequacy and failing to keep up the expected progression gave an experience of not only failing at the goal, but also that they as individuals failed. They felt shame for not doing their duty as a human being and not possessing the qualities that inner ethical dignity represents. The values that prevail here contain the morality and the norms that man has made his own. There is an increased focus on leading a righteous and desirable life and living according to one’s own values and standards. Failure to do so led to shame. Shame arises when people experience their own dignity being threatened. It occurs when the person feels exposed.^36,37^ Eriksson^27^ states that man may experience guilt and lose his own dignity when he fails to take the responsibility that is given to him. This can lead to shame, but this can be a ‘healthy’ shame that can be a source of maturation, growth and increased responsibility.^37,38^ Through the shame, the students gained introspection. They became more familiar with their own vulnerability and shortcomings. They discovered the importance of dignity when it was at stake. Dignity is often described as something we take for granted and do not reflect on until it is threatened. We understand how valuable something is to us, only when we experience that it is gone.^6^ The findings show that new insight is achieved when living in a space of vulnerability between pride and shame. They discovered that life was fragile and that all people are vulnerable. This showed them the importance of taking care of both themselves and others, as if the other were oneself. Vulnerability is a universal human basic condition that can be a resource as it has the ability to create compassion, empathy and responsibility between us humans.^39^

#### Experiences within the external aesthetic dimension of dignity

The findings show that the external aesthetic dimension of dignity was a significant arena where students were able to express their dignity and thus rebuild it. Man needs the external aesthetic dignity to live out the values in action.^6,29^ The external aesthetic dignity is closely linked to the inner ethical, as the standard, the morality and the norms that man has made his own are expressed here.

The external aesthetic dignity also serves as an arena for gaining confirmation from others. The students discovered the dignity through confirmation or lack thereof. Through being seen, heard, believed and taken seriously, dignity becomes noticeable.^6^ The external aesthetic dignity is a bodily dimension.^29^ The students felt both pride and shame bodily. The body experienced both dignity and violation of dignity, and through this they discovered the importance of dignity and confirmation. The findings show different experiences with confirmations in the education. In encounters with patients and fellow students, the students felt confirmed and valuable. Relationships that both conveyed and accepted dignity were important for dignity to be perceived as valid. But the experience of dignity was relative. Contextual circumstances or relationships that did not promote dignity made it difficult for students to experience dignity, like when they were ignored and degraded. Eriksson^40^ emphasizes that to fail to affirm another person’s human dignity is to afflict man with suffering and thus violate his dignity. But also through the experience of violation, the students gained increased insight into dignity. Dignity may be easier to detect when it is not present.^41^ The findings show that experiences with violated dignity made the students aware of the importance of dignity.

A challenge with the concept of dignity is that dignity is often taken for granted and is in danger of losing its meaning.^29^ Some have argued that dignity is a useless concept. The main criticism is that the concept is vague and superfluous.^42^ Our results show that experiences with external aesthetic dignity reveal how dignity can be expressed. Experiences with dignity gave the concept meaning. The external aesthetic dignity is expressed through actions.^6^ Dignity became visible through actions, for example, through observation of how others either promoted or violated dignity. Both the culture in the clinical field and the learning environment are of great importance for how students discover dignity, as well as which role models students acquire.^17,18,22,24^ Clinical supervisors have a key function in creating a learning environment that reflects professional nursing values. The students described some clinical supervisors and nursing teachers that they wanted to resemble, that they respected and trusted. They carefully observed how these people were and how they promoted the dignity of other people. The students also described clinical supervisors and nursing teachers who did not promote dignity. These experiences surprised them and made them reflect on their own interactions. According to von Post,^43^ man is made responsible even when he witnesses that others do not live up to their responsibilities. She found that both nurses and nursing students experienced having their dignity violated by seeing others violating patients. They saw and felt the humiliation as if it were their own. It forced them to stop and reflect and they had to choose whether to be a spectator or to intervene.^43^ Our findings also show that such situations attracted the students’ attention. Although the students lacked the courage to intervene, they turned their attention to what was right and wrong in the various situations. The students’ inner ethical dignity accommodated a standard and morality that they wanted to live by. It became a guiding star for what was the right thing to do. They said they used themselves and their loved ones as a benchmark of what promoted dignity. The inner ethical dignity also draws attention to the standard of others. It contains values such as respect, grandiosity, properness, suitability, restraint and orderliness.^29^ The students did not see these values and distanced themselves from what they called horror examples of violations of patients’ dignity. They lost respect for both nurses and nursing teachers as individuals and for the nursing expertise they represented, when they did not experience the external aesthetic dignity. Combrinck et al.^44^ emphasize the importance of preserving the professional dignity in nursing. If nurses are part of a work environment that does not promote or facilitate dignity, this will not only have an impact on their own experience of dignity, but will also reduce the quality of care patients receive.

## Strengths and limitations

The sample in the study represents a large variety of different types of students, in terms of both age (21–37 years) and personality. Both genders were represented. The variety included in the sample resulted in diversified descriptions of experiences around the discovery of dignity. Also, the various nursing campuses represented in the study had great variation in the content and structure of the education. These factors make the findings transferable to other contexts. At the same time, one must take into account that the experiences the students shared reflect the context the students were in, in terms of the individual campus they represented, the content and focus of the education, learning environment and the personal experiences they had gained through the education. The study was performed in a Norwegian context and within the Norwegian education system, and the findings are characterized by this.

Our preunderstanding has shaped the research process and made understanding possible. Together, we have several years of experience as teachers in nursing education, which constituted extensive knowledge and expertise in the field. The preunderstanding also contained the understanding we already had about dignity. Through the data material, our preunderstanding about dignity was used to build up a greater understanding of the phenomenon. Own preunderstanding was constantly at stake, and at the same time we were conscious of our preunderstanding so that it did not unknowingly affect how we interpreted the data material. The research process had elements of both induction and deduction. We did not provide guidelines for how dignity should be understood, but the theory we had in advance about dignity helped to shape the questions we asked and how we understood the answers we received. In this way, the preunderstanding may have been an obstacle to the openness of what dignity could be and how it could be discovered.

## Conclusion and implication for practice

The study found that the expression and significance of dignity are discovered through experiences. The discovery may happen through experiences in line with the preunderstanding, but experiences that involved a breach with expectations made a greater impact. The students discovered the inner ethical dignity through experiencing vulnerability, pride and shame. When dignity was perceived as threatened, the need to find something that could symbolize dignity increased. They discovered the external aesthetic dignity through actions, both in the form of how they themselves were confirmed or violated, or by observing others who promoted or violated patients’ dignity. This created reflection and awareness of what kind of nurse the students aspired to be.

This article shows how dignity is discovered by students and how the value can be acquired. It is important that the nursing education focuses on students’ experiences of dignity in the education. Increased reflection on the relational aspects of the education must also be themed in the light of the students’ experiences of dignity and how dignity can be expressed in the practice of nursing.
